# Changes in Companion Diagnostic Labelling: Implementation of FDA’s April 2020 Guidance for Industry for In Vitro CDx Labeling for Specific Oncology Therapeutic Groups

**DOI:** 10.1007/s43441-022-00422-z

**Published:** 2022-06-10

**Authors:** Lisa Cooper, Joyce Chen

**Affiliations:** 1 Department of Health Informatics, School of Health Professions, Rutgers, The State University of New Jersey, Stanley S. Bergen Building, Suite 136, 65 Bergen Street, Newark, NJ 07101-1709 USA; 2 Ernest Mario School of Pharmacy, Rutgers, The State University of New Jersey, Piscataway, NJ USA

**Keywords:** Companion diagnostic, Precision medicine, Oncology, Therapeutic class, Labeling

## Abstract

Advanced understanding of the molecular pathways of oncologic diseases has shifted therapeutic treatment development to focus on mechanism of actions targeting specific genomic alterations. These precision medicines are indicated for patient subsets defined by these specific mutations as determined by diagnostic devices approved by the Food and Drug Administration (FDA). The Intended Use section within the companion diagnostic (CDx) labeling has historically specified the therapeutic products for which they have been clinically validated. In April 2020, the FDA reiterated their position that therapeutic class labeling may be used, if appropriate, instead of named products. Labels for FDA approved in vitro CDxs were reviewed to evaluate the implementation of therapeutic class labeling. A total of 47 devices have been approved as of 2 January 2022, of which 3 labels were found to contain therapeutic class labeling: two devices targeting EGFR mutations for the treatment of non-small cell lung cancer (NSCLC), and one targeting BRAF V600E and BRAF/MEK inhibitor combinations for melanoma. Two devices received therapeutic class labeling upon initial approval, while the third implemented the language though a label revision. A total of 25 different indications were identified across the 47 CDx devices, of which 9 (34.6%) were associated with more than 1 CDx device. Implementation of therapeutic class labeling has been slow following the release of the FDA’s April 2020 guidance; however, the potential to incorporate such language into existing and newly approved CDx labels exists. Precedence and manufacturer experience are expected to drive an increase in therapeutic class labeling.

## Introduction

Personalized medicine, now commonly referred to as precision medicine, involves the use of genetic or biomarker information for making treatment decisions [1, 2]. In oncology, traditional approaches in product development and patient treatment decisions were driven by tumor histology, disease stage, and line of therapy [3, 4]; however, recent advanced understanding of the molecular pathology of the disease has progressed the development of drugs whose mechanism of action targets specific genomic alterations [5–8]. These molecular characteristics are linked to the treatment outcomes or predicted therapeutic effects of the product [6, 9, 10]. To ensure use of the targeted therapy in only those patients who are expected to benefit, an in vitro companion diagnostic (CDx) is used to identify the existence or absence of the desired marker. To date, such CDx devices have included immunohistochemistry (IHC) tests, polymerase chain reaction (PCR), in situ hybridization (ISH) devices such as FISH and CISH, as well as other DNA sequencing devices [7].

### CDx Development and Approval

Ideally, development of a CDx should occur as early as possible, essentially in parallel with the development of the drug product, allowing for patient selectivity in clinical trials, often resulting in reduced development time and cost [3, 6, 9, 11]. Co-development of a CDx and a therapeutic product can be challenging and typically requires partnerships between the drug sponsor and in vitro diagnostic (IVD) sponsor. Additionally, it is not always certain that a CDx will be required for a drug product at the start of development; therefore, a CDx may not be introduced until later in the development program [12–14]. In some cases, where the CDx is considered to pose significant risk to study subjects, the sponsor will be required to open an Investigational Device Exemption (IDE) file with the Food and Drug Administration (FDA). Otherwise, a non-significant risk device will be evaluated and monitored by the Institutional Review Board (IRB) along with the clinical study(ies) under the Investigational New Drug (IND) file. Ultimately, before the device can be marketed, it must be validated, demonstrating both analytical (ability to generate accurate and reproduceable results) and clinical (ability to select the appropriate patients for the associated drug) performance [3, 12, 15, 16]. The Phase III clinical study should be designed to provide the necessary clinical performance validation data [6].

Once the necessary validation has been completed for the CDx, the device sponsor may apply for approval, or clearance, by the FDA for market. Most commonly, CDx devices are approved via the Premarket Approval (PMA) pathway, consistent with Class III medical devices. Class III medical devices are defined as those that are intended to be life-supporting or sustaining, or substantially important in preventing impairment of human health, as well as where existing data are insufficient to determine if general controls are adequate to reasonably assure the safety and efficacy of its application. Alternatively, if the level of risk to the patient, based on its intended use, is lower than a Class III device, and it contains adequate controls, the CDx may be cleared for market using the premarket notification submission 510(k) route [7, 17, 18]. Device label revisions for new intended uses, including additions of new drug products for the same intended use, must be approved by the FDA via a supplement to the PMA or new 510(k) [17].

### Labeling

As part of the CDx market application, proposed CDx labeling is provided by the sponsor. CDx labeling should be developed with its corresponding therapeutic product in mind. The therapeutic product label will contain information regarding the need for an approved or cleared IVD companion diagnostic, which is necessary for patient selection or monitoring, dose modification determination, or for response determination or adverse event identification. Importantly, within the therapeutic product label, the brand or manufacturing name of the CDx is not listed, rather the user is directed to the FDA’s “List of Cleared or Approved Companion Diagnostic Devices (In Vitro and Imaging Tools)” WebSite [17]. Conversely, in accordance with the 2014 FDA Guidance for Industry entitled “In Vitro Companion Diagnostic Devices”, the brand name of the corresponding product must be listed in the CDx label’s intended use section. However, the guidance does allow for exception to using brand names as follows: “In some cases, if evidence is sufficient to conclude that the IVD companion diagnostic device is appropriate for use with a class of therapeutic products, the intended use/indications for use should name the therapeutic class, rather than each specific product within the class.” [17] Regardless of this allowance, as noted by Jorgensen (2021), at the end of 2020, a total of 44 CDxs were approved for named drug products, with only one exception [19], the EGFR CDx, Cobas EGFR Mutation Test V2, which received therapeutic group label language as a label update on October 27, 2020 [20].

Despite the FDA’s direction that CDx labeling may specify therapeutic class or group labeling as opposed to naming specific products, the application of this language in approved CDx labels is lacking. The FDA reiterated their position on therapeutic group labeling by releasing an additional guidance as draft in December 2018, and finalized in April 2020 entitled “Developing and Labeling In vitro Companion Diagnostic Devices for a Specific Group of Oncology Therapeutic Products.” [21] Within the guidance, the FDA acknowledges that several approved CDx devices target the same molecular alteration and highlights the benefits of therapeutic group labeling in patient care optimization, such as reducing patient sample burden. It is stressed in the guidance, however, that it is not just a matter of “matching diagnostic targets with therapeutic targets” [21] to determine the viability of therapeutic class labeling. Five points of consideration are provided for CDx sponsors who are assessing the practicality of therapeutic class labeling: (1) Can a therapeutic class be defined? Such oncology products must be “approved for the same indications, including the same molecular alteration(s), such as mutation(s), amplification(s), and fusion(s) for which clinical evidence has been developed with at least one device for the same specimen type for each therapeutic product” [21]; (2) is the mechanism of action of the therapeutic products and the interaction of the products with the biomarker that the CDx identifies defined well enough to determine which products should be included or excluded from the therapeutic class; (3) are there at least two therapeutic products with sufficient clinical data in the defined therapeutic class; (4) is the CDx analytically validated for the range of biomarkers that defined in the indication; and (5) is the CDx clinically validated in the intended disease. Four approved CDx devices for the detection of epidermal growth factor receptor (EGFR) exon 19 deletions or exon 21 (L858R) substitution mutations for patients with non-small cell lung cancer (NSCLC) are used as an example within the guidance which may benefit from therapeutic class labeling. Five named therapeutic products are listed collectively amongst the four CDx labels; however, the number of named products in each CDx label varies from one to four. Therapeutic class labeling would allow use of all five therapeutic products to be used with each CDx, if their labels were updated. It is therefore no surprise that the first CDx to receive approval of therapeutic class labeling following the release of the April 2020 guidance is one of the example devices listed, Cobas EGFR Mutation Test V2, as noted above [21].

Therapeutic class labeling can be sought directly by the CDx sponsor at the time of initial PMA submission or as a PMA supplement or 510(k). Additionally, therapeutic product sponsors can recommend therapeutic class labeling for a CDx to the FDA, who in turn will work with the CDx sponsor if determined appropriate. Regardless, analytical and clinical validation data supporting the broad indication must be provided to the FDA by the CDx sponsor. The level of data required will vary, and those CDx devices which have prior approval or clearance may be able to utilize previously generated data. Alternatively, concordance studies with other previously approved CDx devices for the same population may be conducted of the CDx was not previously approved or cleared. Retrospective sample analysis may also be performed to demonstrate comparable clinical performance, though it should be noted that this type of study would likely require collaboration with the therapeutic product sponsor. The criteria for the analytical and clinical validation data, including study design, should be agreed upon with the FDA prior to submission of the CDx application or supplement, as these will present the biggest challenge in obtaining therapeutic class labeling [21].

It has been more than 18 months since the release of the FDA’s CDx labeling guidance, and it is the intent of this paper to evaluate the implementation of therapeutic class labeling in newly approved and revised in vitro CDx labels.

## Methods

Information for approved CDx devices was obtained from the FDA “List of Cleared or Approved Companion Diagnostic Devices (In Vitro and Imaging Tools)” WebSite accessed 26 September 2021 through 2 January 2022 as well as by following links on the page to the CDx PMA files. If not linked, full CDx label content was retrieved from Devices@FDA.com. Intended Use statements were reviewed for therapeutic group language or specific brand name products. For each CDx device, the data reviewed on the WebSite and PMA files included CDx initial and label supplement approval dates (decision date), number of therapeutic products approved for each CDx, date of initial and subsequent therapeutic product approvals associated with each CDx, number of years between first and second therapeutic product approvals, indication (e.g., NSCLC), and number of gene alterations each CDx identifies. Descriptive statistics were used to summarize numerical data.

## Results

**Fig. 1:**
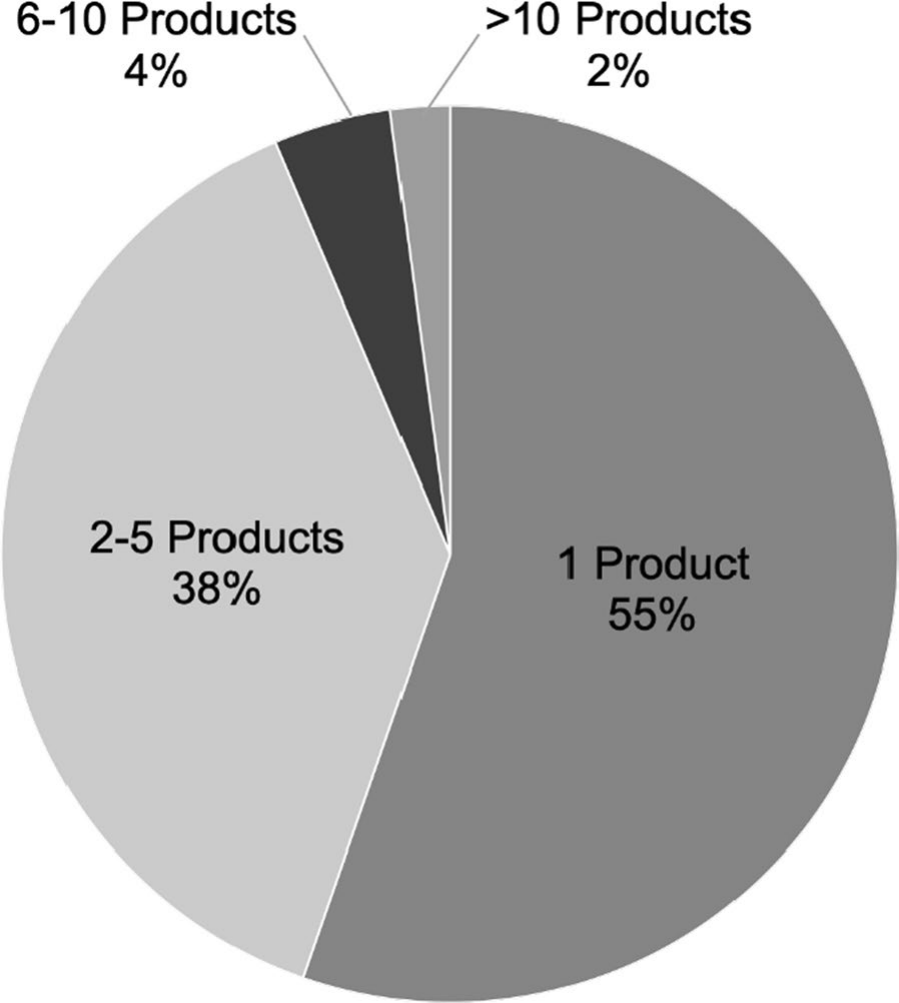
The majority of the 47 approved CDxs list only one named therapeutic product in their labeled Intended Use (55%), followed by labels containing 2–5 named therapeutic products (38%). Few CDx device labels contain 6 or more named therapeutic products

A total of 48 CDx devices were listed on the “List of Cleared or Approved Companion Diagnostic Devices” Website as of 2 January 2022, 47 of which are in vitro diagnostic devices (FerriScan, which is an imaging tool, has been excluded from analysis). The in vitro CDx devices were associated with 47 unique named therapeutic products for which safety and efficacy have been demonstrated, all of which are approved for oncology indications. Of the 47 CDx devices, 26 (55%) were approved for use with 1 named therapeutic product, 18 (38%) for use with 2–5 named therapeutic products, 2 (4%) for use with 6–10 named therapeutic products, and 1 (2%) for use with > 10 named therapeutic products (see Fig. 1). Therapeutic class labeling is not included in the number of named therapeutic products.

Based on initial approval dates of each device, 8 (17.0%) of the 47 CDxs were approved during or after April 2020, with the most recent approval dated 12 October 2021. The earliest CDx approval was 30 December 1997. Of the 26 CDx devices approved for use with 1 named therapeutic product, 5 (19.2%) were approved during or after April 2020. Of the remaining 3 devices of the 8 initially approved during or after April 2020, 2 had 2–5 named therapeutic products in their labels, and 1 had 6–10 named therapeutic products. Of the 21 CDx devices approved for use with 2 or more named therapeutic products, 16 (76.2%) had their named therapeutic products approved in different years (supplement approvals). The average time from initial approval of the CDx to approval for use with a second named therapeutic product was 5.35 years.

Most CDxs (37; 78.7%) were approved for identification of a single targeted genetic mutation or alteration. Five (10.6%) were approved for 2 targeted genetic mutations or alterations, and 1 CDx device was approved for each of the following number of targeted genetic mutations or alterations: 4, 23, 61, 300, and 324 targets.

Only 3 (6.4%) of the identified 47 CDx devices contained therapeutic class labeling, with two devices targeting EGFR mutations for the treatment of NSCLC and one targeting BRAF V600E and BRAF/MEK inhibitor combinations for melanoma. The first therapeutic class labeled CDx approved was the Cobas EGFR Mutation Test V2, which was noted previously. Prior to the therapeutic group labeling of this CDx, three therapeutic products were directly named in the CDx label’s Intended Use section: Tarceva (erlotinib), Tagrisso (osimertinib), and Iressa (gefitinib). The second CDx to incorporate therapeutic group labeling was the ONCO/Reveal Diagnostic Lung and Colon Cancer Assay which included language within the initial label approval on 30 July 2021. The latest device to receive therapeutic class approval is the FoundationOne CDx which, as labeling supplement, was approved on 10 November 2021 for BRAF V600E and BRAF/MEK inhibitor combinations. Initial approval of the FoundationOne CDx, received 30 November 2017, included two named products indicated for BRAF V600E melanoma, Tafinalar (dabrafenib) and Zelboraf (vemurafenib) for BRAF V600E, and two named products, Mekinisth (trametinib) or Cotellic (cobimetrinib) in combination with Zelboraf. Five additional EGFR CDx devices have been approved for NSCLC; however, they did not contain therapeutic class labeling. Table 1 summarizes the two devices with EGFR class labeling, while Table 2 summarizes the EGFR devices without named therapeutic product labeling for NSCLC. Two additional BRAF V600E CDxs are approved for use in melanoma (Table 3) and another two for other indications, *therascreen* BRAF V600E RGQ PCR Kit for colorectal cancer, and Oncomine™ Dx Target Test for NSCLC (not shown).

**Table 1:** EGFR CDxs with NSCLC therapeutic group labeling

	Cobas EGFR mutation test V2	Onco/reveal Dx lung & colon cancer assay (O/RDx-LCCA)
CDx therapeutic group label language	EGFR Tyrosine Kinase Inhibitor (TK1)	EGFR Tyrosine Kinase Inhibitors approved by FDA
Initial approval	05/14/2013	07/30/2021
Specified product(s)	Tagrisso® (Osimertinib)^a^	Erbitux® _(Cetuximab)_ Vectibix® (Panitumumab)^b^
Therapeutic group label approval	10/27/2020 (Supplement)	_07/30/2021 (Initial)_
Targeted genes	EGFR (exon 19 deletions and exon 21 [L858R]); EGFR (T790M, tissue and plasma)	EGFR (exon 19 and exon 21 [L858R]); KRAS (absence of mutations in codons 12 and 13)
Indication(s)	Non-small cell lung cancer	Non-small cell lung cancer, colorectal cancer

^a^Tagrisso® remains a named product for Cobas EGFR Mutation Test V2 for EGFR T790M, plasma positive, tissue negative, or unknown populations

^b^Named products for Onco/Reveal are only for a second indication, colorectal cancer (CRC)

**Table 2:** EGFR CDxs for NSCLC without therapeutic group labeling

	Therascreen EGFR RGQ PCR Kit	FoundationOne CDx	Oncomine Dx target test	FoundationOne liquid CDx
Initial approval	07/12/2013	11/30/2017	6/22/2017	8/26/2020
Specified Product(s)	Gilotrif (Afatinib), Iressa (Gefitinib), Vizimpro (Dacomitinib)	Gilotrif (Afatinib), Iressa (Gefitinib), Tagrisso (Osimertinib), Tarceva (Erlotinib)	Iressa (Gefitinib), Exkivity (Mobocertinib)	Iressa (Gefitinib), Tagrisso (Osimertinib), Tarceva (Erlotinib)
Targeted Genes	Exon 19 deletions, Exon 21 L858R and L861Q, Exon 18 G719X, and Exon 20 S768I	Exon 19 Deletion, Exon 21 L858R; Exon 20 T790M^a^	Exon 19 deletions, Exon 21 L858R, Exon 20 insertions^b^	Exon 19 deletions, Exon 21 L858R substitutions^c^

^a^Additional non-EGFR NSCLC target mutations include: ALK rearrangements, BRAF V600E, and MET single nucleotide variants (SNVs) and indels that lead to MET exon 14 skipping; additional mutations for other indications are included in the intended use

^b^Additional non-EGFR NSCLC target mutations include: BRAF V600E, RET fusions, and ROS1 fusions; additional mutations for other indications are included in the intended use

^c^Additional non-EGFR NSCLC target mutations include: ALK rearrangements and MET single nucleotide variants (SNVs) and indels that lead to MET exon 14 skipping; additional mutations for other indications are included in the intended use

**Table 3:** BRAF V600E CDxs for Melanoma Without Therapeutic Group Labelling

	Cobas 4800 BRAF V600 mutation test	THXID BRAF kit
Initial approval	8/17/2011	5/29/2013
Specified product(s)	Zelboraf (Vemurafenib), Cotellic (Cobimetinib) in combination with Zelboraf (Vemurafenib)	Tafinlar (Dabrafenib); Mekinist (Trametinib), Braftovi (Encorafenib) in combination with Mekinist (Trametinib
Targeted genes	BRAF V600E or V600K	BRAF V600E and V600K
Indication(s)	Melanoma	Melanoma

A total of 25 different indications were identified across the 47 CDx devices, of which 9 (34.6%) were associated with more than 1 CDx device. Table 4 identifies the indications for which more than 1 CDx is associated. In addition, a select number of mutations for indications of interest are highlighted in Tables 5–8. The tables note which named therapeutic products are contained within the approved labeling for the CDxs.

**Table 4:** Indication and associated CDxs

FDA-Approved indication	FDA-approved companion diagnostic
Breast cancer	BRACAnalysis CDx FoundationOne CDx INFORM HER-2/neu PathVysion HER-2 DNA Probe Kit PATHWAY anti-Her2/neu (4B5) Rabbit Monoclonal Primary Antibody InSite Her-2/neu KIT SPOT-LIGHT HER2 CISH Kit Bond Oracle HER2 IHC System HER2 CISH pharmDx Kit INFORM HER2 Dual ISH DNA Probe Cocktail HercepTest HER2 FISH pharmDx Kit Therascreen PIK3CA RGQ PCR Kit VENTANA HER2 Dual ISH DNA Probe Cocktail FoundationOne ® Liquid CDx Ki-67 IHC MIB-1 pharmDx (Dako Omnis)
Non-small cell lung cancer (NSCLC)	Therascreen EGFR RGQ PCR Kit Cobas EGFR Mutation Test v2 PD-L1 IHC 22C3 pharmDx FoundationOne CDx VENTANA ALK (D5F3) CDx Assay Oncomine Dx Target Test Therascreen KRAS RGQ PCR Kit Vysis ALK Break Apart FISH Probe Kit PD-L1 IHC 28–8 pharmDx Guardant360® CDx FoundationOne ® Liquid CDx Onco/Reveal Dx Lung & Colon Cancer Assay (O/RDx-LCCA) Ventana PD-L1 (SP263) Assay
Colorectal Cancer	FoundationOne CDx Praxis Extended RAS Panel Cobas KRAS Mutation Test Therascreen KRAS RGQ PCR Kit Dako EGFR pharmDx Kit Therascreen BRAF V600E RGQ PCR Kit Onco/Reveal Dx Lung & Colon Cancer Assay (O/RDx-LCCA)
Ovarian cancer	BRACAnalysis CDx FoundationOne CDx FoundationFocus CDxBRCA Assay Myriad myChoice CDx FoundationOne ® _Liquid CDx_
Metastatic castrate resistant prostate cancer (mCRPC)	BRACAnalysis CDx FoundationOne CDx FoundationOne ® _Liquid CDx_
Gastric or gastroesophageal junction adenocarcinoma	PD-L1 IHC 22C3 pharmDx HercepTest HER2 FISH pharmDx Kit
Urothelial carcinoma	PD-L1 IHC 22C3 pharmDx Therascreen FGFR RGQ RT-PCR Kit Ventana PD-L1 (SP142) Assay
Acute myeloid leukemia	Abbott RealTime IDH1 Abbott RealTime IDH2 LeukoStrat CDx FLT3 Mutation Assay
Melanoma	THXID BRAF Kit Cobas 4800 BRAF V600 Mutation Test FoundationOne CDx

**Table 5:** CDxs targeting HER2 mutation for breast cancer

FDA-approved companion diagnostic	FDA-approved therapeutic products		
	Herceptin® (trastuzumab)	Kadcyla® (ado-trastuzumab emtansine)	Perjeta® (pertuzumab)
INFORM HER-2/neu	x	–	–
PathVysion HER-2 DNA Probe Kit	x	–	–
PATHWAY anti-Her2/neu (4B5) Rabbit Monoclonal Primary Antibody	x	x	–
InSite Her-2/neu KIT	x	–	–
SPOT-LIGHT HER2 CISH Kit	x	–	–
Bond Oracle HER2 IHC System	x	–	–
HER2 CISH pharmDX Kit	x	–	–
Inform HER2 Dual ISH DNA Probe Cocktail	x	x	–
HerceptTest	x	x	x
HER2 FISH pharmDx Kit	x	x	x
Ventana HER2 Dual ISH DNA Prob	x	–	–

## Discussion

Our analysis shows only recent application of therapeutic class labeling, with implementation limited to the second half of 2021. Three CDx devices, Cobas EGFR Mutation Test V2, ONCO/Reveal Diagnostic Lung and Colon Cancer Assay, and FoundationOne CDx all received approval for therapeutic class labeling following the finalization and release of the April 2020 guidance reiterating the FDA’s position. It is not surprising that two of these devices are for detection of EGFR exon 19 and exon 21 [L858R], given this is the example noted within the FDA guidance as having opportunity for therapeutic class labeling. The Cobas EGFR Mutation Test V2 was the first CDx to receive therapeutic class labeling, achieving the language via a standard 180-day labeling supplement. This was followed by the second EFGR CDx, ONCO/Reveal Diagnostic Lung and Colon Cancer Assay which received therapeutic class labeling upon initial approval. Since release of the guidance and excluding the ONCO/Reveal Diagnostic Lung and Colon Cancer Assay, an additional two EGFR CDx devices were identified, bringing the total EGFR CDx devices appropriate for the same therapeutic class labeling to five (Table 2). The third device, FoundationOne CDx for use with BRAF V600E targeted therapeutics and BRAF/MEK inhibitor combinations, is another example of therapeutic class labeling achieved through a standard 180-day label supplement. FoundationOne CDx, initially approved in 2017, is the third device approved for detection of V600E in melanoma, with prior devices approved in 2013 and 2016 (THXID BRAF Assay Kit and Cobas 4800 BRAF V600 Mutation text, respectively; Table 3). As with the collection of EGFR CDx devices, these BRAF V600E CDx devices identify the same mutation, the therapeutic class has been well established, more than one therapeutic product is associated with the device, and the interaction of the products with the biomarker is well established. It is likely that previously approved EGFR and BRAF V600E devices with named therapeutic devices will also seek to obtain therapeutic class labeling with future label revisions. Additionally, the door has been opened for new devices detecting these mutations for the defined indication to receive therapeutic class labeling upon initial approval.

Four specific genetic mutations, human epidermal growth factor receptor 2 (HER2) for breast cancer, BReast CAncer genes 1 and 2 (BRCA1 and BRCA2) for ovarian cancer, Kirsten Rat Sarcoma Viral Oncogene Homolog (KRAS) wild-type for colorectal cancer, and programed death ligand 1 (PD-L1) expression for NSCLC were explored for therapeutic class labeling potential. HER2 detection includes the most numerous CDx devices for use with 3 named therapeutic products, Herceptin, Kadcyla, and Perjeta, which overlap in several device intended use statements (Table 5). The HER2 CDx device methodologies vary, and include IHC, CISH, FISH, and antibody methods; however, the biomarker is well established within the breast cancer indication associated with the therapeutic products. Additionally, many of the devices received FDA approval several years ago further solidifying their clinical value. Four devices, spanning three therapeutic products, were identified for BRCA1 and BRCA2 for ovarian cancer (Table 6). Like HER2, BRCA1 and BRCA2 are also well-established biomarkers. Two of the BRCA CDx devices are produced by Myriad Genetic Laboratories, Inc. and the other two by Foundation Medicine, Inc. Therefore, Foundation Medicine, Inc., the manufacturer of FoundationOne CDx discussed above for detection of BRAF V600E in patients with melanoma, which is now versed in therapeutic class labeling, could reasonably seek therapeutic class labeling for these additional devices as well. Five CDx devices are approved for KRAS wild type (exons 2, 3, and 4; codons 12 and 13) for use in colorectal cancer (Table 7). With one exception, all devices have intended uses with Erbitux and Vectibix as named therapeutic products (Praxis Extended RAS Panel only includes Vectibix). Two of the devices, FoundationOne CDx and ONCO/Reveal Diagnostic Lung and Colon Cancer Assay, already include therapeutic class labeling for other indications, and a third device, Cobas KRAS Mutation Test, is manufactured by Roche Molecular Systems, Inc., which has therapeutic class labeling for its other device, Cobas EGFR Mutation Test v2. The final group of devices reviewed were the PD-L1 CDxs for use in NSCLC, which utilize antibodies to determine the level of PD-L1 protein expression via IHC staining (Table 8). Each device utilizes a unique clone derived from either mouse or rabbit monoclonal antibodies. Therapeutic group labeling may be more challenging for this collection of devices due to the uniqueness of each device, which may drive the need for more robust clinical validation for each named therapeutic product. Additionally, unlike the other groups reviewed, very little therapeutic product overlap is seen in the intended use statements, adding to the challenge of obtaining therapeutic class labeling.

**Table 6:** CDxs targeting BRCA1 and BRCA2 mutation for ovarian cancer

FDA-approved companion diagnostic	FDA-approved therapeutic products		
	Lynparza® (olaparib)	Rubraca® (rucaparib)	Zejula ® (niraparib)
BRACAnalysis CDx	x	x	–
FoundationFocus CDx BRCA assay	–	x	–
Myriad myChoice CDx	x	–	x
FoundationOne Liquid CDx	–	x	–

**Table 7:** CDxs targeting KRAS wild-type mutation for colorectal cancer

FDA-approved companion diagnostic	FDA-approved therapeutic products	
	Erbitux® (cetuximab)	Vectibix® (panitumumab)
FoundationOne CDx	x	x
Praxis extended RAS panel	–	x
Cobas KRAS mutation test	x	x
Therascreen KRAS RGQ PCR KIT	x	x
Onco/Reveal Dx Lung & colon cancer assay	x	x

**Table 8:** CDxs targeting PD-L1 protein expression for NSCLC

FDA-approved companion diagnostic	FDA-approved therapeutic products			
	Keytruda® (pembrolizumab)	Libtayo® (cemiplimab-rwlc)	Tecentriq® (atezolizumab)	Opdivo® (Nivolumab) + Yervoy® (ipilimumab)
PD-L1 IHC 22C3 pharmDx	x	x	–	–
Ventana PD-L1 (SP142) assay	–	–	x	–
PD-L1 IHC 28–8 pharmDX	–	–	–	x
VENTANA PD-L1 (SP263) assay	–	–	x	–

Limitations in the analysis of the above mutations is acknowledged, as similarity of the CDx devices were only evaluated by review of the labeled intended use and mutation targets. Further assessment of cut-offs, filters, or other design features to determine identification of the same patient population was not performed. Such evaluation would need to be completed to further determine appropriateness of broad therapeutic class labeling.

## Conclusion

The three CDx devices which have achieved therapeutic class labeling provide precedence for other CDx devices to obtain labeling that is not restrictive to named therapeutic products. Target mutations for which more than one device is approved for the same intended use and which are associated with well-established therapeutic products present the best opportunity for therapeutic class labeling. Those devices which are developed by experienced manufacturers may be the quickest to revise current labeling as well as to obtain therapeutic class labeling at initial approval. Early and frequent communication with the FDA is recommended to determine if the CDx is a candidate for and the appropriate level of data needed to support therapeutic labeling. Overall, the shift to therapeutic class labeling has begun, and we are likely to see a progressive movement to adopt this more inclusive language in the next few years.
